# Chronic heat stress in tropical urban informal settlements

**DOI:** 10.1016/j.isci.2021.103248

**Published:** 2021-11-10

**Authors:** Emma E. Ramsay, Genie M. Fleming, Peter A. Faber, S. Fiona Barker, Rohan Sweeney, Ruzka R. Taruc, Steven L. Chown, Grant A. Duffy

**Affiliations:** 1 School of Biological Sciences, Monash University, Clayton, VIC 3800, Australia; 2 School of Public Health and Preventive Medicine, Monash University, Clayton, VIC 3004, Australia; 3 Centre for Health Economics, Monash Business School, Monash University, Clayton, VIC 3145, Australia; 4 Public Health Faculty, Hasanuddin University, Makassar, Indonesia

**Keywords:** Weathering, Environmental health, Environmental issues

## Abstract

The health and economic impacts of extreme heat on humans are especially pronounced in populations without the means to adapt. We deployed a sensor network across 12 informal settlements in Makassar, Indonesia to measure the thermal environment that people experience inside and outside their homes. We calculated two metrics to assess the magnitude and frequency of heat stress conditions, wet bulb temperature and wet bulb globe temperature, and compared our *in situ* data to that collected by weather stations. We found that informal settlement residents experience chronic heat stress conditions, which are underestimated by weather stations. Wet bulb temperatures approached the uppermost limits of human survivability, and wet bulb globe temperatures regularly exceeded recommended physical activity thresholds, both in houses and outdoors. Under a warming climate, a growing number of people living informally will face potentially severe impacts from heat stress that have likely been previously overlooked or underestimated.

## Highlights

• Chronic heat stress was observed outdoors and in houses in informal settlements
• Wet bulb temperature reached 30.5°C, approaching the limit of survival
• Heat stress regularly exceeded recommended physical activity thresholds
• Heat stress in informal settlements was underestimated by weather stations

## Graphical abstract

**Figure:**
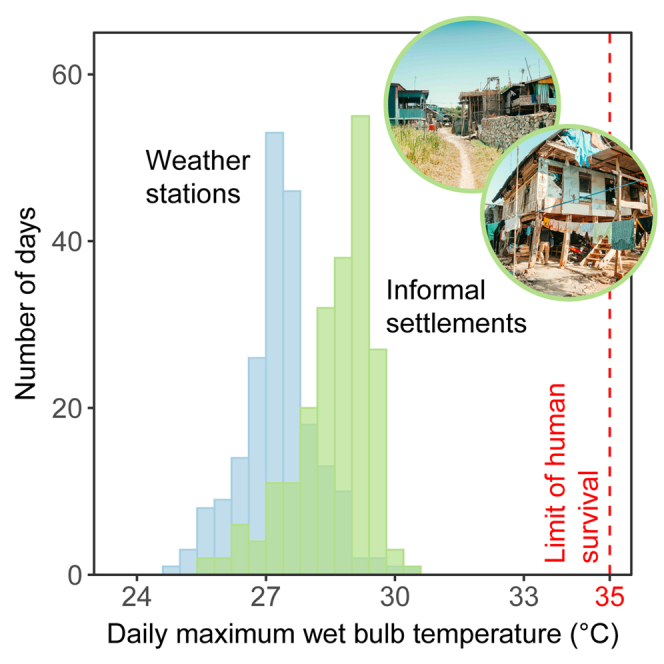

## Introduction

Extreme heat stress, arising from combinations of high temperature and high humidity, has adverse health, wellbeing and economic impacts on all humans. Reported health impacts range from heat stroke and exacerbation of existing illnesses, to excess mortality (Mora et al., 2017; Dosio et al., 2018). Heat stress also leads to reduced productivity and work capacity, which can cause personal financial hardship and have large-scale economic consequences (Lucas et al., 2014; Orlov et al., 2020). As global climate change progresses, human populations will be exposed to warmer average temperatures and more frequent, intense and extended extreme weather events (Dosio et al., 2018; IPCC, 2021). Under a moderate greenhouse gas emissions scenario (Representative Concentration Pathway [RCP] 4.5), more than 50% of the world's population could be exposed to potentially deadly combinations of temperature and humidity by 2100 (Mora et al., 2017), alongside losses to global Gross Domestic Product of between 0.5% and 2.4% (low and high emissions scenarios, RCPs 2.6 and 8.5, respectively) as a result of heat-induced reductions in work capacity (Orlov et al., 2020). Given that global greenhouse gas emissions are currently tracking RCP 8.5, the highest emissions scenario used by the IPCC (Schwalm et al., 2020), heat stress is likely to have far-reaching and ongoing impacts.

Exposure to extreme heat disproportionately affects vulnerable populations in low latitude cities because the tropics will experience the largest increase in the frequency of extreme heat events (Coffel et al., 2018), and those living informally may lack the resources to invest in mitigation strategies. One billion people live in informal settlements worldwide, which generally lack secure land tenure and basic services and infrastructure, and are thus vulnerable to multiple impacts of climate change (Satterthwaite et al., 2020), including heat stress. In addition, informal settlements generally comprise low-quality housing, and residents often depend on informal employment in labour-intensive roles for their livelihoods (Ezeh et al., 2017). These inherent characteristics of informal settlements, along with a large existing health burden, mean that residents are highly vulnerable to heat stress, and lack adequate refuge and financial security if extreme heat reduces work capacity (Pasquini et al., 2020).

Human survival under extreme heat conditions has a physiological upper limit of 35°C wet bulb temperature (TW; Sherwood and Huber, 2010). Extreme heat in tropical and sub-tropical regions was previously predicted to exceed this threshold in the mid to late 21^st^ century (Im et al., 2017); however, a recent analysis of global weather station data has revealed that the daily maximum TW has already surpassed 35°C in South-Asia and the Middle East, with frequent observations of TW above 31°C in these and other locations worldwide (Raymond et al., 2020). Extreme wet bulb temperatures across the tropics are also expected to increase in near-perfect unison with regional mean warming (Zhang et al., 2021). Yet, extreme heat may still be underestimated in topographically complex and/or data-scarce areas, such as urban informal settlements (Scott et al., 2017; Baruti et al., 2019; Banerjee et al., 2021), where regional meteorological models may not capture the intricacies of temperature and humidity variation and extremes at a local scale and *in situ* environmental monitoring is lacking (Coffel et al., 2018; Wang et al., 2019). Indoor temperatures are rarely examined in informal settlements (but see Mukhopadhyay et al., 2021 and Wilby et al., 2021) and it may be assumed that adaptations, such as the use of air-conditioning, will be practiced (Biardeau et al., 2019), even though mitigation of this kind is expensive and often unavailable to informal settlement households (Satterthwaite et al., 2020). These shortcomings and assumptions likely result in vulnerable populations with limited capacity to adapt being overlooked in broader scale heat stress exposure assessments (Scovronick et al., 2015; Green et al., 2019). The extent and magnitude of heat stress exposure in informal settlements could, therefore, be underestimated (Baruti et al., 2019). Information to understand the extent of this problem is required to ensure that informal settlement residents are not left behind in the global action being taken to address the Sustainable Development Goals (Ezeh et al., 2017).

Here, we address these knowledge gaps by explicitly measuring the magnitude and frequency of extreme and chronic heat stress experienced both in houses and outdoors by people living in urban informal settlements in South-East Asia. By measuring heat stress at a local scale, we aim to understand the full extent to which heat stress, and its potential health and economic impacts, is likely to be a problem for people living in informal settlements.

**Figure 1:**
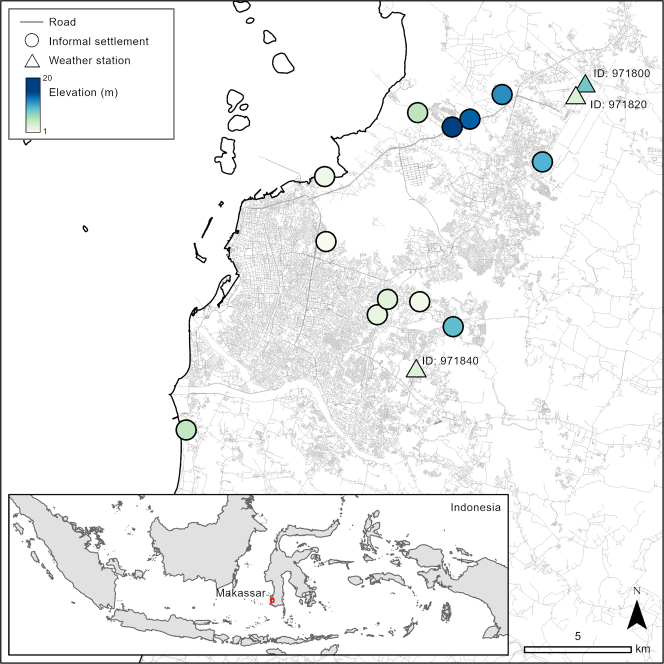
Map of Makassar, Indonesia, showing locations and elevation of informal settlements where loggers were deployed, and weather stations Elevation data is from the Shuttle Radar Topography Mission (SRTM; Farr et al., 2007; USGS EROS Archive: https:://doi.org/10.5066/F7PR7TFT), means were calculated for each settlement and point values extracted for each weather station. Shapefiles of Indonesia sourced from the GADM database version 3.4 (www.gadm.org). Shapefile of roads sourced from OpenStreetMap (https://www.openstreetmap.org).

**Figure 2:**
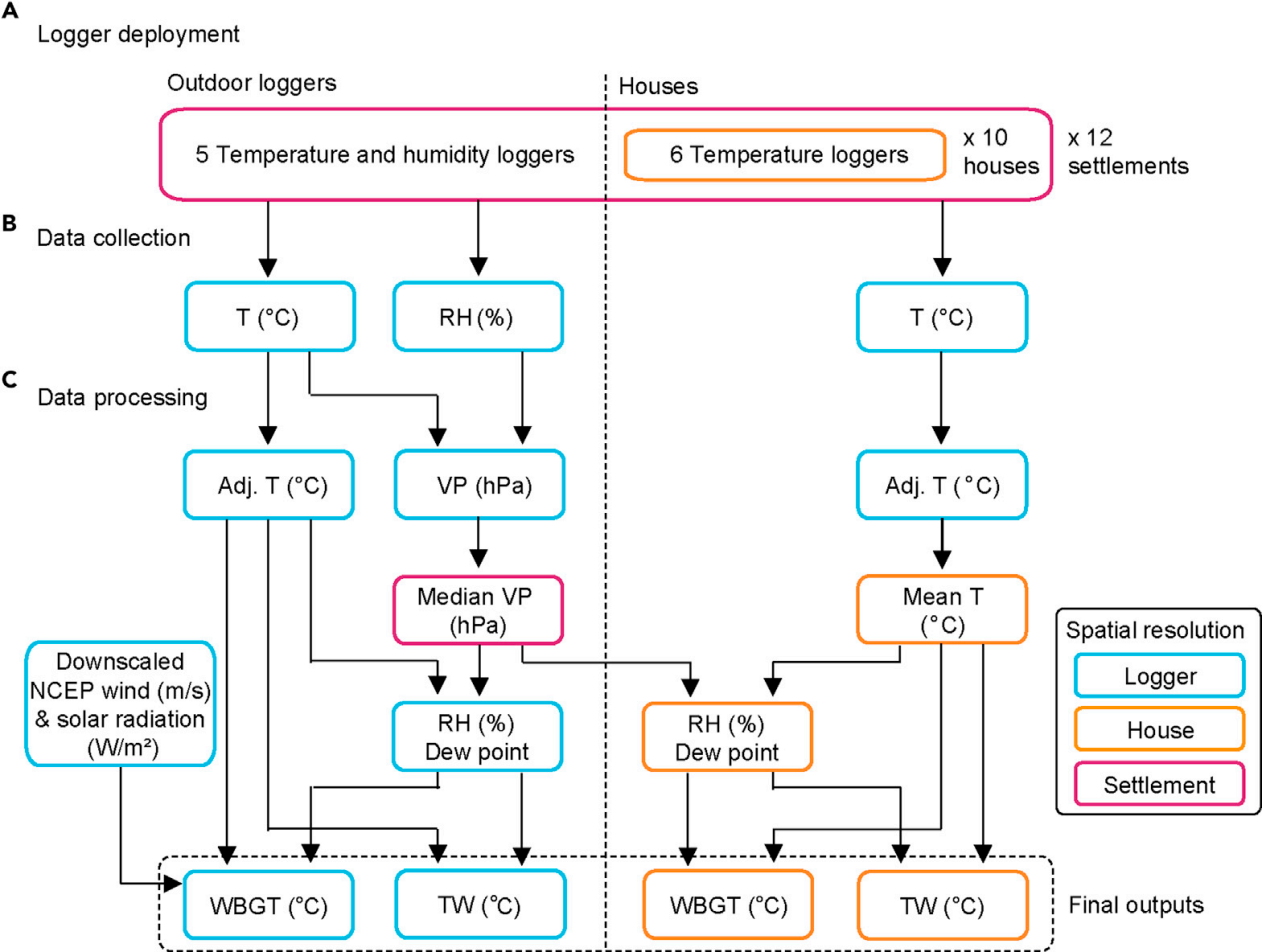
Flowchart summarizing logger deployment, data collection and data processing in informal settlements (A and B) 65 loggers were deployed across each of the 12 settlements studied (B) recording either temperature (T) or temperature and relative humidity (RH). (C) Wet bulb temperature (TW) was calculated using adjusted temperature (adj. T) values and relative humidity derived from settlement-level vapor pressure (VP). Wind speed and solar radiation data downscaled from the National Centers for Environmental Prediction (NCEP) were used in addition to logger-derived variables to calculate wet bulb globe temperature (WBGT) for each outdoor logger.

We collected *in situ* measurements of hourly temperature and humidity outdoors, along with temperature measurements in 119 houses, in 12 urban informal settlements in Makassar (ind: *Kota Makassar*; mak: *ᨀᨚᨈ ᨆᨀᨔᨑ*), a coastal city on the island of Sulawesi, Indonesia (Figure 1). These settlements represent more than 30% of Indonesia's urban population (United Nations, 2020) and some of the 370 million people living informally in East and South-East Asia (United Nations, 2021). We calculated two measures important for assessing thermal conditions and their impacts on humans (Figure 2). Wet bulb temperature (TW) is calculated from air temperature and humidity and is most commonly used to quantify or forecast extreme conditions (Raymond et al., 2020). Wet bulb globe temperature (WBGT) includes the effects of solar radiation and wind and is widely used in occupational research to estimate heat stress at different activity levels (Parsons, 2006). We computed daily maximum TW and identified where WBGTs exceeded recommended activity thresholds for extended time periods. We compared these data to the same calculations made using local weather station data.

## Results and discussion

**Figure 3:**
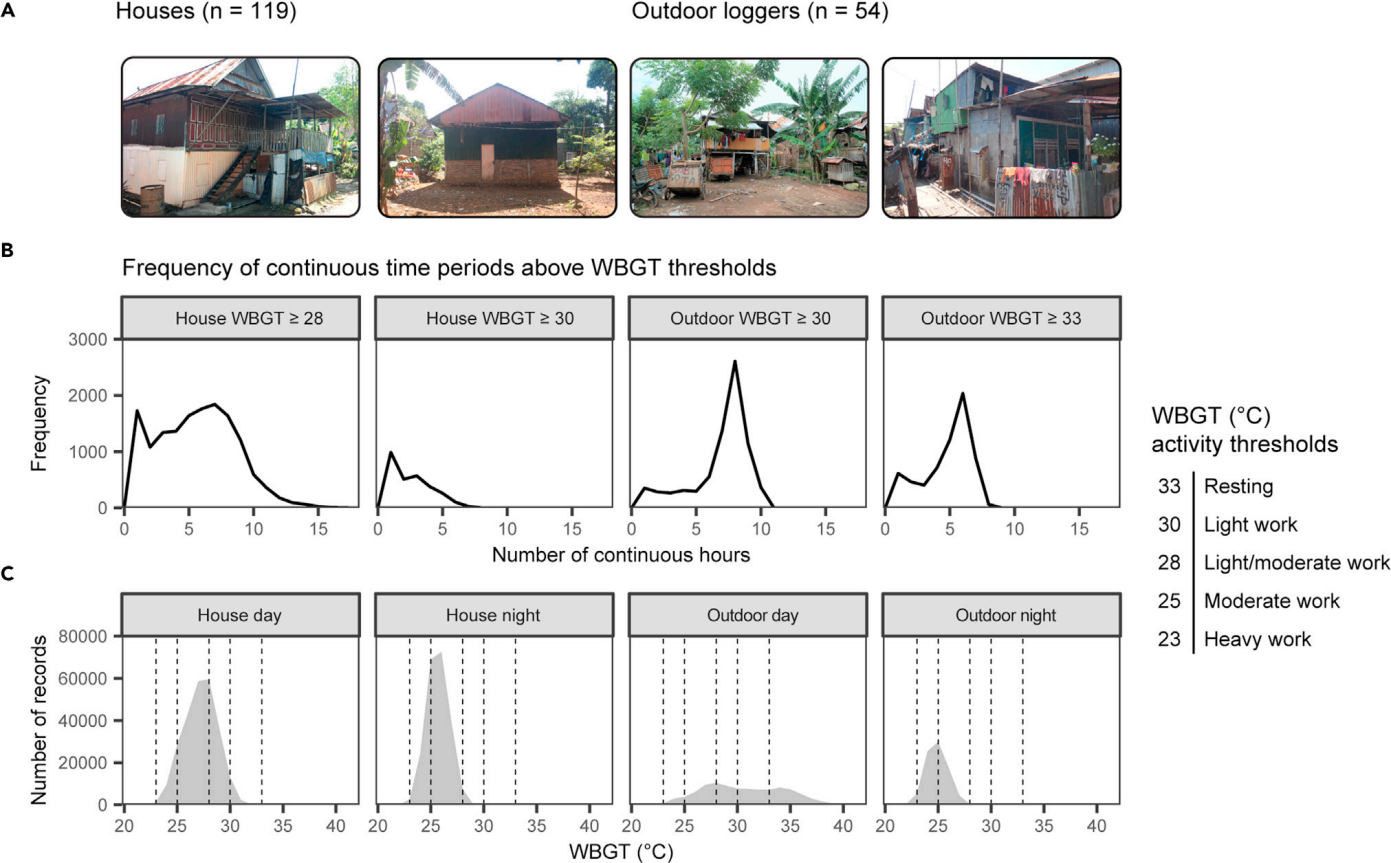
WBGT in houses and outdoors in informal settlements in Makassar, Indonesia (A) Representative photos of informal settlements in Makassar, Indonesia. (B) Frequency of continuous time periods above WBGT activity thresholds. (C) Frequency of records of WBGT in houses (left) and outdoors (right). WBGT activity thresholds are from Parsons (2006) including heavy work (metabolic load >260 Wm^−2^), moderate work (200 < metabolic load <260 Wm^−2^), moderate/light work (130 Wm^−2^ < metabolic load <200 Wm^−2^), light work (65 Wm^−2^ < metabolic load <130 Wm^−2^) and resting (metabolic load <65 Wm^−2^). Photograph credit L - R: E. E. Ramsay, E. E. Ramsay, RISE Consortium, B. C. Josey.

Across all 12 informal settlements WBGTs (calculated as detailed in Figure 2) regularly exceeded recommended human activity thresholds for extended periods (Figure 3). Overall, 79.6% of all data records surpassed a WBGT threshold of 25°C (Figure 3C), above which heat effects on health and productivity have been observed in most occupations (Flouris et al., 2018). Outdoors, WBGTs exceeded the threshold for light work (WBGT ≥ 30°C; Parsons, 2006) for up to 11 consecutive hours, and over the ∼7-month sampling period there were 6,337 instances where this threshold was exceeded for more than 5 consecutive hours (Figure 3B). Outdoor WBGTs exceeded the threshold for resting (WBGT ≥ 33°C; Parsons, 2006) for up to 9 hours at a time (Figure 3B). WBGTs in houses did not reach the same extremes as those outdoors during the day (Figure 3), but still provided little relief from thermally stressful conditions and frequently exceeded activity thresholds. For example, house WBGTs regularly exceeded the threshold for light/moderate work (WBGT ≥ 28°C; Parsons, 2006) for up to 18 hours at a time (Figure 3). At night, when people are more likely to be spending time indoors, WBGT in houses was warmer than WBGT outdoors, remaining as high as 29.4°C.

**Figure 4:**
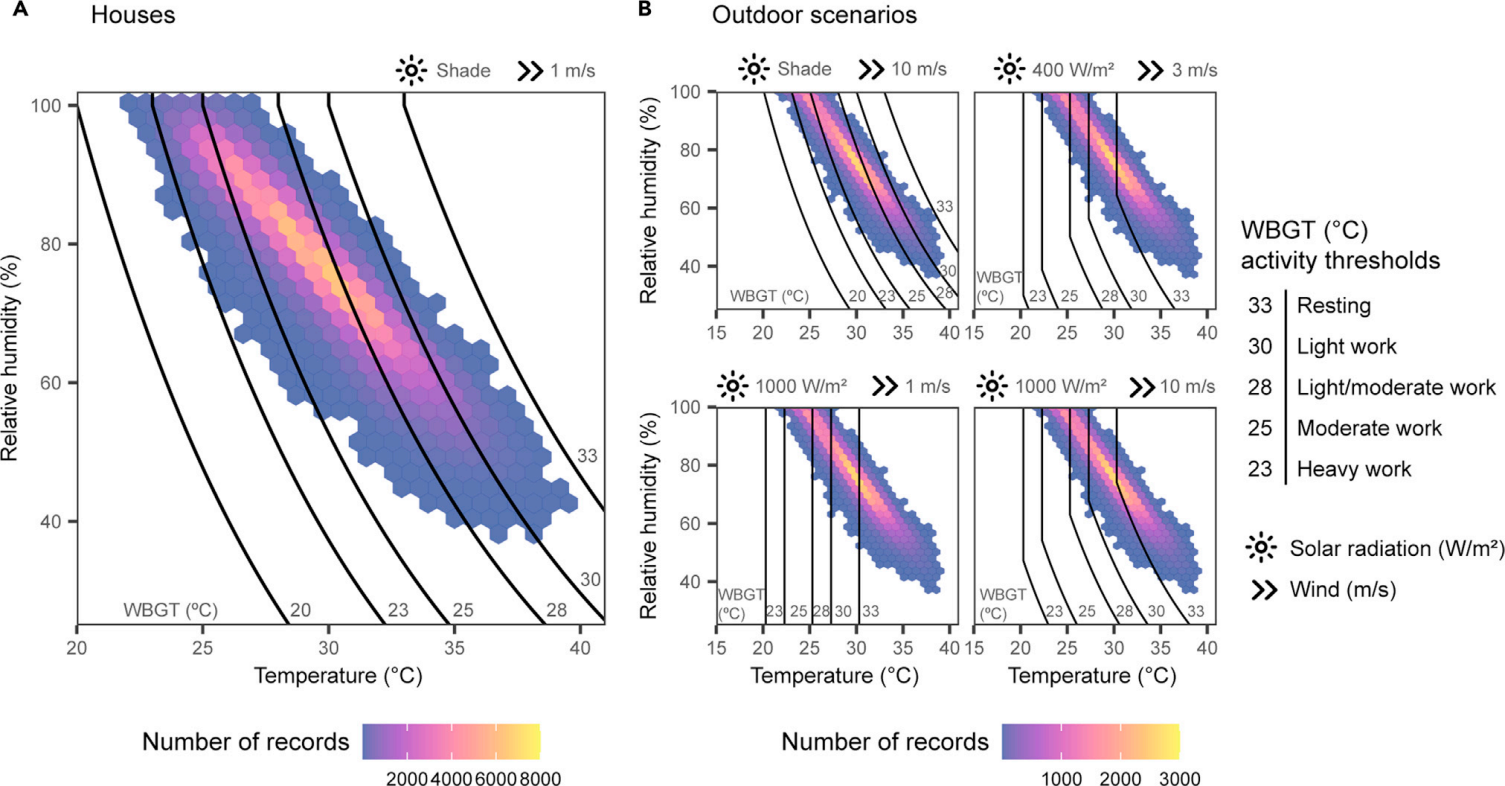
Frequency of WBGT records under different heat stress scenarios (A) Frequency of records of WBGT calculated from relative humidity (%) and temperature (°C) in houses. (B) Frequency of records of WBGT calculated from relative humidity (%), temperature (°C) and different wind and solar radiation scenarios for day time data collected by outdoor loggers. Contour lines show WBGT activity thresholds from Parsons (2006) including heavy work (metabolic load >260 Wm^−2^), moderate work (200 < metabolic load <260 Wm^−2^), moderate/light work (130 Wm^−2^ < metabolic load <200 Wm^−2^), light work (65 Wm^−2^ < metabolic load <130 Wm^−2^) and resting (metabolic load <65 Wm^−2^).

Day time WBGT scenarios calculated using different combinations of solar radiation and wind illustrate that heat stress is likely unavoidable in these informal settlements (Figure 4). For example, under high solar radiation (1000 W/m^2^), the cooling effects of increasing wind speed from 1 m/s to 10 m/s do little to lessen WBGTs (Figure 4B). Even under a moderate scenario (400 W/m^2^ and 3 m/s), WBGTs frequently exceeded the recommended threshold for resting. Therefore, after accounting for variation in estimates of solar radiation and wind, it is almost certain that people living in these settlements experience frequent and excessive heat exposure, which can lead to higher rates of heat illness and mortality (Lucas et al., 2014). For example, a meta-analysis of occupational heat strain found that 15% of individuals who frequently work in heat stress conditions had kidney disease or acute kidney injury (Flouris et al., 2018). Although previous literature has argued for greater thermal adaptation in human populations from the tropics, much of the human response to heat loads depends on thermal exposure patterns, individual variation and the general wellbeing of individuals (Taylor, 2014). This variability makes it difficult to ascertain the extent to which acclimation could reduce the impacts of heat stress on informal settlement dwellers, or if acclimation affords any measurable protection, especially because informal settlement dwellers are among the most vulnerable of urban dwellers to health and wellbeing stressors of other kinds (Ezeh et al., 2017).

**Figure 5:**
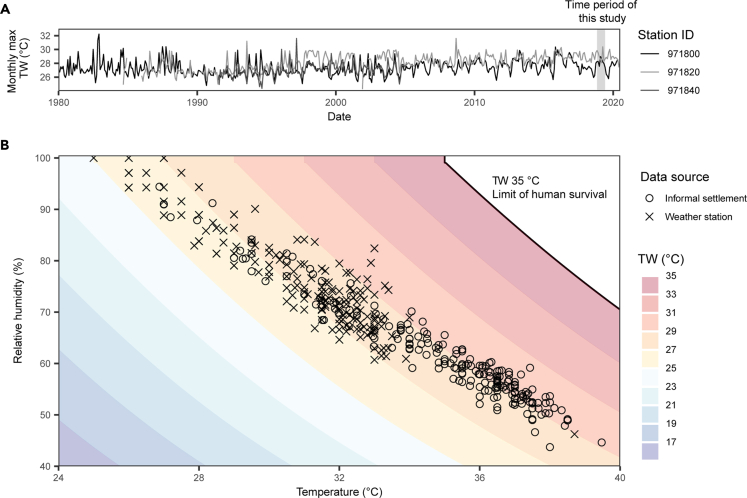
TWs in informal settlements and at weather stations (A) Monthly maximum TW (°C) from a full-time series of available weather station data in Makassar, Indonesia showing the time period of this study (note that not all weather stations cover the full time period). (B) Daily maximum TW, from combinations of relative humidity (%) and temperature (°C), in informal settlements and from weather station data, showing the limit of human survival (TW 35°C; Sherwood and Huber, 2010).

In the informal settlements studied here, such potentially debilitating conditions are the norm. We recorded absolute maximum TWs in informal settlements of 30.2°C in houses and 30.5°C outdoors (Figure 5B). Excess mortality has been reported at TWs at and below these conditions. For example, the 2003 European heatwave reached TWs no greater than 28°C and contributed to thousands of deaths (Coffel et al., 2018; Raymond et al., 2020) and the 2015 heatwave in India reached an absolute maximum TW of 31°C with similar outcomes (Im et al., 2017; van Oldenborgh et al., 2018). Of the 212 days captured by our observations, 166 days (78.3%) had maximum TWs over 28°C (Figure 5B). The period of recording covered Makassar's wet season, when humidity is at its highest seasonally, but the 2018/19 season does not stand out as extraordinary in the full weather station time series (Figure 5A, 1980 to 2020). More extreme heat stress, which approaches the absolute limit of human survivability (TW of 35°C; Sherwood and Huber, 2010), is expected over the next century. Extreme TWs in the tropics are expected to increase in line with mean warming (Zhang et al., 2021), leading to likely several-fold increases in the frequency of extreme TWs by 2080 (Coffel et al., 2018). Widespread, long-term *in situ* monitoring will, therefore, prove essential for monitoring and responding to future extreme weather events and their impacts on informal settlement environments and residents.

Daily maximum TW in informal settlements was on average 1.3°C higher than that measured by weather stations, and tended to arise from warmer temperatures coupled with lower humidity (Figure 5). Temperature in urban areas can be elevated by upwards of several degrees because of the urban heat island effect, which is driven by increases in anthropogenic materials which absorb and retain heat, compounded by reduced vegetation cover which both increases temperature and reduces humidity (Sharmin et al., 2015; Bai et al., 2017). The magnitude of urban heat islands has also been found to be greater in densely packed, low-income neighborhoods, such as informal settlements (Jacobs et al., 2019). As we demonstrate here, this local-scale effect of urbanization is unlikely to be captured by weather stations (See Figure S2 for example time series comparing temperature measured by weather stations with the range captured in informal settlements), unless they are located within such settlements or similar surroundings. Therefore, TW and WBGT calculated using weather station data, may underestimate localised thermal stress. This has implications for forecasts which suggest that limiting warming to 1.5°C would keep the majority of the tropics from surpassing the 35°C limit of human survivability (Sherwood and Huber, 2010; Zhang et al., 2021). If extreme TWs are already underestimated by upwards of 1°C, as our results suggest, then the likelihood of TWs surpassing 35°C, particularly in dense urban areas, such as informal settlements, could be much more likely than currently forecast.

Typically, housing is seen as an adaptive mechanism to provide refuge from heat. However, in the informal settlements we measured, conditions in houses provided some, but not extensive relief from extreme WBGTs outdoors (Figure 3). Furthermore, asset-ownership surveys indicated that few residents had the adaptive capacity to respond to extreme heat in their own homes. Only 4.4% of households had air-conditioning, well below the regional and national averages (22.8% and 16.9% respectively, National Population and Family Planning Board BKKBN; Statistics Indonesia BPS; Ministry of Health Kemenkes; ICF, 2017). Electric fan ownership (89.4% of households) was higher than the regional average (80.4%; National Population and Family Planning Board BKKBN; Statistics Indonesia BPS; Ministry of Health Kemenkes; ICF, 2017). However, the efficacy of evaporative cooling (for example through the use of an electric fan) for personal thermoregulation is severely reduced in humid environments (Hanna and Tait, 2015). The focus of upgrading programs in informal settlements is usually centered on water and sanitation, but we suggest that mitigation of thermal stress should receive greater attention (Pasquini et al., 2020). Given that heat adaptation strategies are unlikely to be available to all households, community-level mitigation and urban interventions will be essential to mitigate heat stress effects. Community-level strategies may include urban greening and modifications to the built environment (Sharmin et al., 2015; Nutkiewicz et al., 2018) that mitigate urban heat island effects, and communal heat shelters to provide refuge in otherwise dense and hot settlements (Satterthwaite et al., 2020).

### Conclusions

Overall, we have shown that informal settlements experience consistently high TWs and WBGTs, which regularly exceed activity thresholds for extended periods of time, even for resting and light work (Parsons, 2006). This heat stress approached the uppermost limits of human thermoregulatory capacity. Furthermore, broader scale studies that use weather station data have likely overlooked or underestimated the magnitude and extent of localized heat stress in informal settlements (Scott et al., 2017; Coffel et al., 2018) and extreme heat will be exacerbated by future warming (Zhang et al., 2021). Housing provides some respite from the most extreme conditions, but residents are still exposed to chronic heat stress within their homes. Given that there is little to no capacity for residents to seek refuge from extreme heat in their residences or adopt technologies that allow adaptation, heat stress is a potentially severe challenge to the large and growing number of vulnerable people that live in informal settlements worldwide. Under a warming climate, heat stress in this large and vulnerable demographic should become a priority in sustainable development projects (Satterthwaite et al., 2020).

### Limitations of the study

Our study took place over the 2018/19 wet season. Long term monitoring is required to understand seasonal variation and long-term trends in heat stress, particularly under extreme conditions such as heatwaves.

We downscaled solar radiation and wind data to calculate WBGT. *In situ* measurements of these variables would strengthen our results.

## STAR★Methods

### Key resources table

**Table:** 

REAGENT or RESOURCE	SOURCE	IDENTIFIER
**Deposited data**		

NOAA National Center for Environmental Information Integrated Surface Database	NOAA National Centers for Environmental Information, 2001	https://www.ncei.noaa.gov/products/land-based-station/integrated-surface-database
iButton raw data	This paper	Bridges: https://doi.org/10.26180/5f571be86c8bd
iButton processed data	This paper	Bridges: https://doi.org/10.26180/5f571be86c8bd

**Software and algorithms**		

R statistical software	R Core Team, 2019	https://www.r-project.org/
*HeatStress* Package	Casanueva, 2019	https://github.com/anacv/HeatStress/
*NicheMapR* Package	Kearney et al., 2020	https://mrke.github.io/
*dplyr* Package	Wickham et al., 2020	https://github.com/tidyverse/dplyr
ggplot2 Package	Wickham, 2016	https://ggplot2.tidyverse.org/
Wet bulb temperature algorithm	Stull, 2011	Stull, 2011
Wet bulb globe temperature algorithm (Liljegren)	Liljegren et al., 2008	Liljegren et al., 2008
Wet bulb globe temperature algorithm (Bernard)	Bernard and Pourmoghani, 1999	Bernard and Pourmoghani, 1999
Code to process iButton data and calculate heat stress metrics	This paper	Bridges: https://doi.org/10.26180/16689385

### Resource availability

#### Lead contact

Further information and requests for resources should be directed to and will be fulfilled by the lead contact, Emma Ramsay (emma.ramsay1@monash.edu).

#### Materials availability

This study did not generate new unique materials.

### Experimental model and subject details

#### Human subjects

Household surveys were undertaken in 593 households (532 adult-female respondents, 61 adult-male respondents, median age 39.6), across the 12 informal settlements. The survey was preferentially directed to the adult female head of household. Ethics review and approval was provided by participating universities and local IRBs, including Monash University Human Research Ethics Committee (Melbourne, Australia; protocol 9396) and the Ministry of Research, Technology and Higher Education Ethics Committee of Medical Research at the Faculty of Medicine, Universitas Hasanuddin (Makassar, Indonesia; protocol UH18020110). The RISE program is a randomised control trial registered on the Australian New Zealand Clinical Trials Registry (ANZCTR) (Trial ID: ACTRN12618000633280). All study settlements provided consent for participation in the RISE project and households provided informed consent for participation in the household surveys.

### Method details

#### Logger deployment

We recorded *in situ* measurements of air temperature and relative humidity from a network of iButton data loggers (Thermochrons DS1921G and Hygrochrons DS1923; Maxim Integrated, San Jose, CA) deployed in 12 urban informal settlements in Makassar, Indonesia. Settlements were selected to be a part of the broader Revitalising Informal Settlements and their Environments (RISE) study and are geographically spread across Makassar (Leder et al., 2021). These settlements comprise dense housing (30–100 houses within each settlement), experience environmental stressors, including flooding and contamination and represent the most vulnerable populations of the city (Leder et al., 2021). We focus here on data collected during the wet season from the 1^st^ November 2018 to 31^st^ May 2019, when year-round warm temperatures in the tropics are compounded by high humidity and are, therefore, a significant source of thermal stress.

The data loggers used have an accuracy of ±1°C between –30°C and 70°C (Thermochrons DS1921G) and ±0.5°C between –10°C and 65°C and ±5% from 0–100% relative humidity (Hygrochrons DS1923). To ensure that data loggers were providing reliable data they were tested in temperature-controlled rooms prior to deployment. Approximately 65 data loggers were deployed per settlement, both in houses and outdoors, totalling 778 successfully deployed data loggers across all 12 settlements. Five loggers were deployed outdoors at each settlement at a height of approximately two metres in custom solar radiation shields (modified from Scott et al., 2017), recording temperature and humidity hourly. Ten houses at each settlement were randomly selected to each host six temperature-only loggers, deployed in solar radiation shields where necessary. Three pairs of loggers (with one sampling hourly, one two-hourly) were deployed in and around (e.g. on verandas or underneath elevated houses) each house (with the exception of one house in Settlement E (Table S1), where the household requested that only two pairs of loggers be deployed).

#### Data collection

Data were downloaded quarterly by a local field team. Logger attrition and data loss (resulting from logger loss, removal, or failure and read errors in the field) was unavoidable in this ever-changing urban environment and access to houses at the time of download was not always possible. Thus, data from all deployed loggers were not recoverable for all time periods (Table S1). Additionally, only periods where both temperature and humidity data were retrieved for a settlement were included in analyses.

In parallel with environmental data collection, a total of 593 households, across the 12 informal settlements, completed a baseline household survey between November 2018 and January 2019 (Leder et al., 2021; French et al., 2021). This baseline survey was used to collect household level data, including household composition, environmental risks, housing quality, tenure, water and sanitation services, solid waste practices, and household assets. Survey questions were translated from English to Bahasa Indonesia and back translated to English to ensure accuracy and consistency. Key phrases or concepts were also translated into two local dialects (Bugis and Makassarese) to assist respondents whose first language was not Bahasa Indonesia. Surveys were completed as face-to-face interviews in the respondent's home, with trained local field surveyors using tablets to electronically record survey responses. Data on household assets, including air/conditioning and electric fans, were collected using the question and associated Showcard “Look at the Showcard and tell me if anyone in the household owns any of the following: computer/laptop; mobile/smart phone; access to internet; electricity; radio/stereo; television/video/DVD; refrigerator; washing machine; water pump; air conditioner; bank account; gas/electric stove; bicycle/pedicab; motorcycle/motorised pedicab; car/truck/van/minibus; outboard motor; electric fan; hand tractor/lawn mower; water tank; generator”. The Showcard included images and text for each listed item.

#### Data processing

Despite the use of solar radiation shields, solar radiation can inflate temperature in urban environments (Terando et al., 2017). To account for this, we calculated the 95^th^ percentile of all temperature measurements at each timepoint (following Braschler et al., 2020), separately for houses and outdoor loggers, and adjusted all data points above this down to the 95^th^ percentile (Adj. T; See Figure S1 for comparison of data adjusted to the 90^th^ and 95^th^ percentiles with unadjusted data). For each house, we then calculated hourly mean adjusted temperature. As humidity data were only collected outdoors, we calculated median vapour pressure from relative humidity for each settlement at each time point. We then recalculated relative humidity, separately for each outdoor logger and house, from settlement-wide median vapour pressure and adjusted temperature values. This was then used to calculate dew point. Thus, although our analyses necessitated the assumption that vapour pressure was homogenous across each settlement, temperature at each outdoor logger and house is still accounted for in the calculation of relative humidity and dew point.

We then calculated hourly TW from adjusted temperature and new relative humidity (RH) using the Stull (2011) formula which is accurate for air temperatures between −20°C and 50°C and relative humidity between 5% and 99% (where relative humidity was over 99%, the wet bulb temperature was assumed to be equal to the dry bulb air temperature) with mean absolute error less than 0.3°C at standard sea-level barometric pressure of 1013 hPa (the elevation of informal settlements and weather stations ranged between 1 and 19.25 m above sea-level):

$$TW\;\left({}^{\circ}C\right)=adj.\;T\;\times \;\text{arctan}\;\left(0.151977{\left(RH+8.313659\right)}^{1/2}\right)+\arctan\;\left(adj.\;T+RH\right)-\arctan\;\left(RH-1.676331\right)+0.00391838\times {\left(RH\right)}^{\frac{3}{2}}\;\times \arctan\;\left(0.023101\times RH\right)-\;4.686035$$

We also calculated WBGT for houses under the assumption of completely shaded (i.e. indoor) conditionsfha with a wind speed of 1 ms^−1^, from temperature and dew point, using the model from Bernard and Pourmoghani (1999) implemented in the R *HeatStress* package (Casanueva, 2019). For outdoor loggers we calculated WBGT using the model from Liljegren et al. (2008) which incorporates solar radiation and wind as well as temperature and dew point. For each outdoor logger we estimated hourly time series of wind and solar radiation, downscaled from the National Centers for Environmental Prediction 6-hourly reanalysis dataset using the *micro_ncep* function in the *NicheMapR* R package (Kearney et al., 2020). Because it is difficult to accurately estimate these variables in a complex urban environment we calculated median monthly values at each time point to provide a reasonable estimate of what a person might experience in unshaded outdoor conditions. To illustrate the potential range of WBGT we repeated these calculations for day time data collected by outdoor loggers under different combinations of wind and solar radiation. These scenarios were calculated at midday and at the mean latitude and longitude of the informal settlements. Scenarios ranged from a low heat stress scenario in full shade with high wind speed (shade and 10 m/s), a moderate scenario with both moderate solar radiation and wind (400 W/m^2^ and 3 m/s), a high heat stress scenario with high solar radiation and low wind speed (1000 W/m^2^ and 1 m/s) and a second high heat stress scenario with high solar radiation but also high wind to potentially alleviate heat stress (1000 W/m^2^ and 10 m/s).

To enable comparison between *in situ* and weather station data, we downloaded all available weather station data from the Integrated Surface Database (NOAA National Centers for Environmental Information, 2001) for 3 stations (Station IDs: 971800, 971840, 971820) in Makassar between 1^st^ January 1980 and 28^th^ June 2020. These data typically have a temporal resolution between 1 and 3 hours and where there was more than one record per hour, we calculated means. From temperature and dew point measurements marked as “passed all quality control checks”, including checks for data continuity, consistency and extreme values (Vose et al., 2011), we calculated TW, using the same methods described for the informal settlement data.

Finally, we computed daily maximum TW for informal settlements and weather stations (where there were equal highest we took the record occurring earliest in the day) and continuous time periods above WBGT thresholds (Parsons, 2006) in houses and for outdoor loggers, allowing for a 2-hour gap between continuous records to account for some periods where only 2-hourly data were recovered. WBGT thresholds were based on the International Organisation for Standardisation (ISO) Heat Stress Standard and provide reference WBGTs at given levels of work, for an acclimatised person to maintain an internal body temperature below 38°C (Parsons, 2006).

All data processing and analysis were performed in R Statistical Software (R Core Team, 2019), using the *HeatStress* (Casanueva, 2019), *NicheMapR* (Kearney et al., 2020), *dplyr* (Wickham et al., 2020) and *ggplot2* package (Wickham, 2016).

## Data Availability

All original code has been deposited at Bridges and is publicly available as of the date of publication. DOIs are listed in the key resources table. The raw and processed iButton data have been deposited at Bridges but remain under embargo until 2 years, after the completion of E.E.R's PhD. Reasonable requests for earlier data access will be made following negotiations with all authors. DOIs are listed in the key resources table. Any additional information required to reanalyse the data reported in this paper is available from the lead contact upon request.
