# Supplementary material for: Chronic heat stress in tropical urban informal settlements
Source: iScience. 2021 Nov 10;24(11):103248. doi: 10.1016/j.isci.2021.103248 (PMC8609203; doi:10.1016/j.isci.2021.103248)
Supplement: Document S1. Figures S1 and S2 and Table S1 [file mmc1.pdf]

iScience, Volume *24*

Supplemental information

Chronic heat stress in tropical urban informal settlements

Emma E. Ramsay, Genie M. Fleming, Peter A. Faber, S. Fiona Barker, Rohan Sweeney, Ruzka R. Taruc, Steven L. Chown, and Grant A. Duffy

Supplementary information

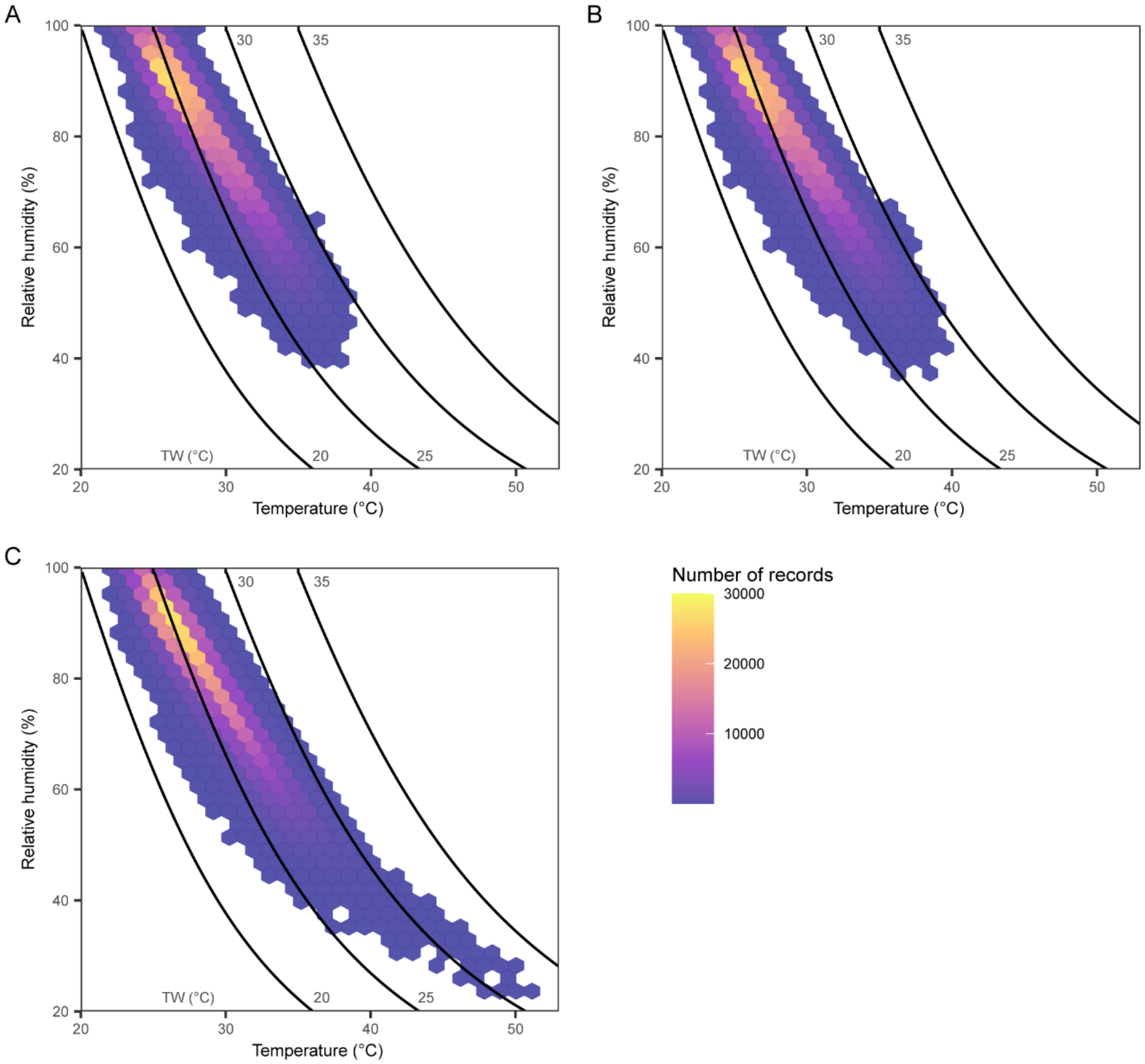

Figure S1. Frequency of records of TW in informal settlements calculated from relative humidity and bounded temperature measurements, related to STAR methods.

(A) Temperature measurements upper-bounded at the 90th percentile, (B) temperature measurements upper-bounded at the 95th percentile and (C) unadjusted temperature measurements.

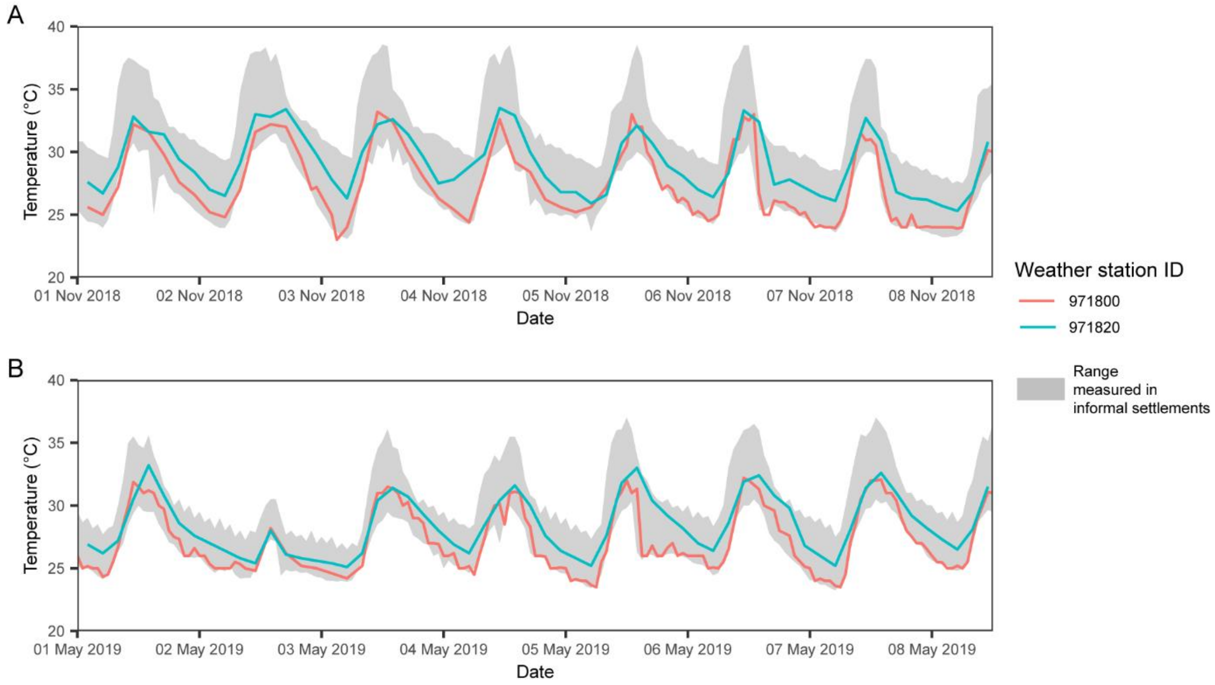

Figure S2. Example time series of data loggers and weather stations, related to Figure 5. (A) Timeseries from November 2018 and (B) May 2019 of temperature data collected by weather stations and range of data captured by *in situ* loggers in informal settlements over the same time period.

Table S1. Number of houses and outdoor data loggers included in analysis for each settlement among three sampling periods between 1st November 2018 and 31st May 2019, related to STAR methods.

Periods where only temperature data were retrieved, and not humidity, were excluded.

Settlement		Sampling period 1 (1st Nov 2018 – mid-Jan 2019)	Sampling period 2 (mid-Jan 2019 – mid-Apr 2019)	Sampling period 3 (mid-Apr 2019 – 31st May 2019)
A	Houses	9	9	7
	Outdoor loggers	5	4	1
B	Houses	10	7	6
	Outdoor loggers	5	3	2
C	Houses	10	9	7
	Outdoor loggers	3	4	1
D	Houses	10	8	0
	Outdoor loggers	5	5	0
E	Houses	10	10	10
	Outdoor loggers	4	3	3
F	Houses	10	10	9
	Outdoor loggers	5	2	1
G	Houses	10	10	10
	Outdoor loggers	4	4	2
H	Houses	10	9	0
	Outdoor loggers	4	3	0
I	Houses	10	0	7
	Outdoor loggers	5	0	1
J	Houses	10	10	0
	Outdoor loggers	5	5	0
K	Houses	10	9	0
	Outdoor loggers	3	3	0
L	Houses	10	7	0
	Outdoor loggers	3	3	0
**TOTAL**	**Houses**	**119**	**98**	**56**
	**Outdoor loggers**	**51**	**39**	**11**
